# Financial toxicity experienced by rural Australian families with chronic kidney disease

**DOI:** 10.1111/nep.14192

**Published:** 2023-06-07

**Authors:** Nicole Scholes‐Robertson, Katrina Blazek, Allison Tong, Talia Gutman, Jonathan C. Craig, Beverley M. Essue, Kirsten Howard, Germaine Wong, Martin Howell

**Affiliations:** ^1^ Sydney School of Public Health The University of Sydney Sydney New South Wales Australia; ^2^ Centre for Kidney Research The Children's Hospital at Westmead Sydney New South Wales Australia; ^3^ College of Medicine and Public Health Flinders University Adelaide South Australia Australia; ^4^ Institute of Health Policy, Management and Evaluation University of Toronto Toronto Ontario Canada; ^5^ Menzies Centre for Health Policy and Economics, Sydney School of Public Health The University of Sydney Sydney New South Wales Australia

**Keywords:** access, chronic kidney disease, financial toxicity, out‐of‐pocket costs, quality of life

## Abstract

**Aim:**

Chronic kidney disease (CKD) and its treatment places a financial burden on healthcare systems and households worldwide, yet little is known of its financial impact, on those who reside in rural settings. We aimed to quantify the financial impacts and out‐of‐pocket expenditure experienced by adult rural patients with CKD in Australia.

**Methods:**

A web based structured survey was completed between November 2020 and January 2021. English speaking participants over 18 years of age, diagnosed with CKD stages 3–5, those receiving dialysis or with a kidney transplant, who lived in a rural location in Australia.

**Results:**

In total 77 (69% completion rate) participated. The mean out of pocket expenses were 5056 AUD annually (excluding private health insurance costs), 78% of households experienced financial hardship with 54% classified as experiencing financial catastrophe (out‐of‐pocket expenditure greater than 10% of household income). Mean distances to access health services for all rural and remote classifications was greater than 50 kilometres for specialist nephrology services and greater than 300 kilometres for transplanting centres. Relocation for a period greater than 3 months to access care was experienced by 24% of participants.

**Conclusion:**

Rural households experience considerable financial hardship due to out‐of‐pocket costs in accessing treatment for CKD and other health‐related care, raising concerns about equity in Australia, a high‐income country with universal healthcare.

## Summary at a Glance

Australian rural patients requiring dialysis or kidney transplantation experience out‐of‐pocket costs that place high levels of stress on their families. Despite living in a Country with universal health insurance, expenses experienced by patients and their families, especially in areas of transportation and travel, can place undue burden on them and impact their ability to access treatment options.

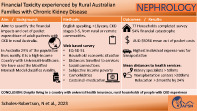

## 1 INTRODUCTION

Globally the prevalence of chronic kidney disease (CKD) is estimated to be 10% and rising, with less than half of the people requiring dialysis and kidney transplantation being able to access kidney replacement therapy.^1^, ^2^ The economic burden on health systems for provision of kidney replacement therapy varies greatly between countries but is estimated to be 2%–7% of health budgets.^3^, ^4^, ^5^ At an individual level, access to kidney replacement therapy is influenced by many factors, but one crucial dimension of access is the availability of affordable healthcare.^6^, ^7^ Globally much of CKD treatment is financed out‐of‐pocket, leaving treatment unaffordable and out of reach to many, particularly those who do not have access to subsidized healthcare or where the level of subsidy is inadequate.^8^, ^9^

In 2018, the United Nations calculated that 45% of the world's population lived in rural settings and that 80% of people living below the international poverty line live in rural areas.^10^ People living in rural areas experience greater socioeconomic disadvantage, a disproportionate burden of CKD, particularly in remote regions, reduced availability and access to public infrastructure and geographical barriers that all contribute to late referral to kidney specialist services and poorer outcomes.^11^, ^12^, ^13^, ^14^, ^15^, ^16^ Socioeconomic factors are a major driver of health outcomes, affecting not only access to healthcare, but also the ability to adhere to treatments across many chronic illnesses, as well as impacting the ability to access treatment compared to people living in urban areas.^15^, ^17^, ^18^, ^19^, ^20^, ^21^

Dialysis and kidney transplantation are essential lifesaving treatments for those with Stage 5 CKD.^3^ The treatment of choice for most patients is kidney transplantation due to improved survival rates and quality of life experienced when compared with dialysis. Kidney transplantation is, in most circumstances, also cost saving compared to dialysis.^22^, ^23^, ^24^, ^25^ Rural patients requiring a kidney transplant have additional costs to those in urban areas because of transportation and accommodation costs to attend and stay at centralized transplantation centres. Much of these costs are covered by the patient due to inadequacy or absence of subsidies.^26^, ^27^

The term financial toxicity is commonly used in cancer care to refer to the quantitative financial consequences (financial hardship) combined with the qualitative or subjective financial concerns patients have whilst receiving treatment.^28^, ^29^, ^30^ In this paper we quantify the financial hardship and presence of catastrophic health expenditure (out‐of‐pocket expenditure greater than 10% of household income) associated with accessing dialysis and kidney transplantation. In a previous paper discussing rural patients' perspectives on access to kidney replacement therapy we discuss the subjective components of financial toxicity including ‘compounding economic consequences’ and ‘unrelenting financial strain’ faced by rural patients.^16^

People with chronic illnesses face higher household out‐of‐pocket healthcare expenditures,^31^ however, the financial toxicity experienced by patients with CKD living in a rural setting in a high income country with universal public health insurance scheme such as Australia is largely unknown. Rural patients with CKD have reported profound economic consequences, depletion of income/leave and difficulty coping with unexpected expenses in accessing kidney replacement therapy.^16^ The aims of this study were to assess the financial toxicity experienced by people with CKD who reside in rural settings in Australia.

## 2 METHODS

### 2.1 Setting and participants

English speaking participants over the age of 18 years, diagnosed with CKD stages 3–5, patients receiving dialysis or those with a kidney transplant, who resided in a rural/remote location in Australia were eligible. A flyer was used to advertise the study on social media platforms including Facebook and Twitter (through the peak consumer group Kidney Health Australia), which contained an anonymous link to an online survey and a participant information sheet. If participants provided consent but required assistance to complete the survey, they were able to email researchers (NSR and MH) and assistance was provided over the phone, and in some locations face to face assistance was provided. The survey was available online from November 2020 until January 2021. This study received ethics approval from the University of Sydney (HREC 2020/009), and this included all patient facing documents and recruitment strategy. Box 1 provides further context regarding the Australian healthcare setting and the financial responsibilities of patients. This was a targeted survey.

BOX 1 **Context**

In Australia, approximately 29% (7 million) of the population live in rural areas.^32^ Australia has a universal public health insurance scheme, but for some medical appointments, tests, and medications, there can be a gap payment or co‐payment required to be paid by the patient.^8^ The purpose of introducing Medicare to the Australian health system was to bring equity to accessing health services.^33^ Medications are subsidized through the Pharmaceutical Benefits Scheme (PBS) with a maximum co‐payment for each prescription filled, the value of which varies according to the welfare status of the patient.^34^ In addition to Australia's PBS and universal healthcare, in 2009 a range of health initiatives were introduced to better support Indigenous Peoples by 7 addressing disparities in access to health and education, commonly known as the ‘Closing the Gap’ policy and funding that assists to reduce out of pocket expenses for Indigenous people.^35^ Private health insurance is a personal choice and there are strong tax incentives to encourage uptake. Private insurance covers services not covered by Medicare^36^ and does not cover co‐payments.

### 2.2 The study questionnaire

The web‐based survey was based on a previous questionnaire used in a metropolitan CKD population and updated to address rural specific issues based on a systematic review, a qualitative interview study on the perspectives of patients on access to dialysis and transplant and the first authors' (NSR) lived experience as a rural patient requiring kidney replacement therapy.^16^, ^26^ The survey included questions from existing validated tools and the quality‐of‐life instrument EQ‐5D‐5L.^16^, ^26^, ^37^, ^38^ The survey collected demographics, medical history including comorbidities and disabilities, distances travelled to medical services, quality of life, household economic situations, and the numbers of social connections. In addition to objective questions about income and socioeconomic status, a validated question regarding subjective income poverty was asked: ‘Given your current needs and financial responsibilities, would you say that you/your family are^32^ Prosperous^8^; Reasonably comfortable^33^; Just getting along^34^; Poor; or Very poor?’.^39^

### 2.3 Measures and definitions

For the online survey we defined the completion rate as the number of people who finished the survey compared to the number who agreed to participate.^40^ The financial hardship experienced by rural households was assessed by self‐reported out‐of‐pocket expenses on all medical and health related costs, excluding private health insurance premiums and through questions on experiences of financial hardship, which is defined as the inability to afford basic costs of living and medical care, and the utilization of dissaving strategies (e.g., drawing on savings or superannuation to pay for basic costs of living and medical care) in the past 3 months.^37^ The term financial toxicity refers to the quantitative financial consequences (financial hardship) combined with the qualitative or subjective financial concerns patients have whilst receiving treatment.^28^, ^29^, ^30^

Out‐of‐pocket expenses captured direct medical costs including medication and co‐ or gap payments and non‐medical costs such as transportation and accommodation related to management and treatment of CKD. Participants were asked about any out‐of‐pocket expenses in the previous three‐month period for the following instances: medications; specialist and general practitioner appointments; hospital charges; medical tests and imaging; ambulance fees; medical equipment and supplies; home modifications; special dietary food; transportation and accommodation costs for medical purposes.^37^, ^41^ The equivalized household income (referred to as ‘Income’ from here forwards) was calculated using the Organization for Economic Cooperation and Development's equivalence scales.^42^ Financial catastrophe is defined as an out‐of‐pocket expenditure greater than 10% of household income.^43^

All participants completed the EQ‐5D‐5L, a two‐part self‐reported utility based‐quality of life measure that includes a five‐dimensional health profile, with each dimension having 5 response levels and a self‐rated global valuation of perceived health using a visual analogue scale where perfect health is scored at 100 and worst possible health is scored at 0.^44^ The utility value on a scale of 0 (death) to 1(full health) was calculated for each participant using an Australian tariff.^45^

Reporting of distances travelled one way (kilometres) was obtained as a part of the survey for the categories of general practitioner (primary health physician), nephrologist, dialysis centre or home training centre and transplantation centre if appropriate. The Modified Monash Model classifies a location as metropolitan, regional centre, large, medium, or small rural town, remote, or very remote.^32^ Rural is referred to in this study as being inclusive of the Modified Monash Categories (MMC) 2–7, with MMC 1 being metropolitan^46^

### 2.4 Analysis

We undertook a descriptive analysis, with differences between groups compared using one‐way anova for continuous variables and a fisher exact test for categorical variables. Statistical analyses were undertaken using IBM SPSS Statistics 26 and R using the tableone package.^47^, ^48^

## 3 RESULTS

### 3.1 Recruitment and participant characteristics

In total, 77 participants completed 100% of the survey (completion rate of 69%) and their characteristics can be seen in Table 1. Overall, there were 11 participants with stage 3–4 CKD, 42 participants were receiving dialysis, and 44 were kidney transplant recipients. Of those currently receiving dialysis, 50% (*n* = 21) were attending satellite units for Haemodialysis and 16% (*n* = 7) were currently on peritoneal dialysis. The mean age of respondents (patient or family member) was 52 years (standard deviation (*SD*) = 13 years), 47 were female (60%) and 8 (7%) identified as Indigenous. A total of 55 (71%) were married or living with a partner, with 45 (58%) reporting that they were currently not working, retired or unable to work due to medical reasons. Seventy‐six (83%) participants were from or lived close to rural towns (MMC2‐5), 4 (4%) were from remote communities (MMC6) and 3 (3%) resided in very remote communities (MMC7). Twenty four percent (*n* = 22) of rural participants had experienced relocation away from their home for a period of greater than 3 months to access necessary treatment for CKD. Participants reported having 0 to 6 comorbidities. The majority reported 2 or more comorbidities with only 5 (6%) and 14 (16%) experiencing no or 1. Of the remainder 48 (56%) reported 1 to 2, 28 (33%) 3 to 4 and 5 (6%) reported 5 to 6 comorbidities. Hypertension (53%, *n* = 59) and depression/anxiety (30%, *n* = 33) were the most reported comorbidities. Almost half of the participants (38%) had current private health insurance.

**TABLE 1:** Participant characteristics overall and by hardship status^b^.

*N* = 77 total completed surveys	Overall^a^	No hardship	Hardship	*p*‐value
Gender (%)				.71
Female	47 (60.3)	10 (66.7)	30 (56.6)	
Male	30 (38.5)	5 (33.3)	22 (41.0	
Age (mean (SD))	52 (13.0)	55.7 (15.7)	50.2 (11.8)	.15
Ethnicity: Aboriginal/torres strait Islander	8 (7.1)	0 (0.0)	7 (12.5)	.34
Marital status (%)				.69
Married/defacto	55 (70.5)	12 (80.0)	37 (69.8)	
Separated/widowed/single/divorced	22 (28.6)	3 (20.0)	15 (28.3)	
Prefer not to say	1 (1.3)	0 (0.0)	1 (1.9)	
Current CKD stage (%)				.46
CKD not requiring dialysis	11 (11.3)	1 (6.7)	6 (10.7)	
Dialysis	42 (43.3)	6 (40.0)	30 (53.6)	
Transplant	44 (45.4)	8 (53.3)	20 (35.7)	
Dialysis modality (%)				.69
Peritoneal dialysis	7 (15.9)	0 (0.0)	5 (16.1)	
Home haemodialysis	16 (36.4)	2 (50.0)	13 (41.9)	
Hospital/satellite	21 (47.7)	2 (50.0)	13 (41.9)	
Education level achieved (%)				.02
Primary school only	2 (2.6)	0 (0.0)	1 (1.9)	
Year 9/10 school certificate	14 (17.9)	1 (6.7)	11 (20.8)	
Year 12	6 (7.7)	1 (6.7)	5 (9.4)	
TAFE (trade certificate)	37 (47.4)	4 (26.7)	27 (50.9)	
University bachelor's degree	19 (24.4)	9 (60.0)	9 (17.0)	
Employment (%)				.49
Fulltime work	16 (20.5)	3 (20.0)	13 (24.5)	
Part time work	4 (5.1)	1 (6.7)	2 (3.8)	
Part time due to medical reasons	5 (6.4)	0 (0.0)	4 (7.5)	
Not currently working	21 (26.9)		16 (30.2)	
Not currently working/medical reasons	1 (1.3)	0 (0.0)	1 (1.9)	
Retired	11 (14.1)	3 (20.0)	7 (13.2)	
Retired due to medial reasons	12 (15.4)	3 (20.0)	7 (13.2)	
Student	8 (10.3)	3 (20.0)	3 (5.7)	
Household income (%)				.28
Under 20 000 AUD	1 (1.3)	1 (7.1)	0 (0.0)	
20 000 AUD–39 999 AUD	33 (42.9)	5 (35.7)	24 (44.4)	
40 000 AUD–59 999 AUD	14 (18.2)	2 (14.3)	12 (22.2)	
60 000 AUD–79 999 AUD	13 (16.9)	2 (14.3)	10 (18.5)	
80 000 AUD–99 999 AUD	6 (7.8)	0 (0.0)	2 (3.7)	
100 000 AUD or over	7 (9.1)	3 (21.4)	4 (7.4)	
Did not know/prefer not to say	3 (3.9)	1 (7.1)	2 (3.7)	
Modified Monash remoteness category				.16
MMC1 metropolitan	9 (9.8)	2 (13.3)	4 (7.4)	
MMC2 regional centres	6 (6.5)	1 (6.7)	4 (7.4)	
MMC3 large rural towns	20 (21.7)	3 (20.0)	14 (25.9)	
MMC4 medium rural towns	17 (18.5)	1 (6.7)	10 (18.5)	
MMC5 small rural towns	33 (35.9)	5 (33.3)	20 (37.0)	
MMC6 remote communities	4 (4.3)	1 (6.7)	2 (3.7)	
MMC7 very remote communities	3 (3.3)	2 (13.3)	0 (0.0)	
Comorbidities (%)				
Cancer	11 (9.8)	3 (20.0)	5 (8.9)	.46
Cardiovascular disease	22 (19.6)	3 (20.0)	16 (28.6)	.74
Diabetes	25 (22.3)	3 (20.0)	17 (30.4)	.64
Hypertension	59 (52.7)	10 (66.7)	38 (67.9)	1
High cholesterol	27 (24.1)	4 (26.7)	16 (28.6)	1
Chronic lung disease	5 (4.5)	1 (6.7)	2 (3.6)	1
Depression and anxiety	33 (29.5)	5 (33.3)	22 (39.3)	.90
Total comorbidities (Mean (SD))	2.41 (1.3)	2.20 (1.2)	2.39 (1.3)	.61
Number of comorbidities (%)				
0	5 (5.8)	0 (0)	5 (8.9)	.56
1	14 (16.3)	3 (20)	7 (12.5)	.43
2	34 (39.5)	9 (60)	21 (37.5)	.15
3 to 4	28 (32.6)	2 (13.3)	20 (35.7)	.12
5 to 6	5 (5.8)	1 (6.7)	3 (5.3)	1
Social interactions				.03
0	24 (30.0)	1 (6.7)	20 (36.4)	
1 to 3	42 (52.5)	9 (60.0)	29 (52.7)	
4 to 6	13 (16.2)	4 (26.7)	6 (10.9)	
>6	1 (1.2)	1 (6.7)	0 (0.0)	
Accommodation				.004
Own home with mortgage	32 (42.7)	6 (40.0)	22 (43.1)	
Own home without mortgage	21 (28.0)	9 (60.0)	9 (17.6)	
Private rental	10 (13.3)	0 (0.0)	9 (17.6)	
Rented social housing	12 (16.0)	0 (0.0)	11 (21.6)	
Relocation >3 months (YES %)	22 (23.7)	2 (13.3)	14 (25.5)	.52
EQ5D‐5L (mean (SD))	0.60 (0.03)	0.61 (0.10)	0.59 (0.04)	.83
CKD not requiring dialysis	0.68 (0.27)	0.86 (0.31)	0.55 (0.13)	.35
Dialysis	0.54 (0.34)	0.49 (0.13)	0.54 (0.06)	.73
Transplant	0.65 (0.25)	0.66 (0.11)	0.67 (0.07)	.94

*Note*: *p* value is difference between hardship and non‐hardship group.

^a^ *n* is variable due to varying number of respondents answering each question.

^b^ Hardship status was determined through inability to pay and/or dissaving's strategies.

The average EQ5D quality of life utility score reported was 0.60 (95% CI 0.54–0.67) where a score of 1 represents full health and a score less than 0 indicates a health state worse than death, the lowest scores were in those receiving dialysis at 0.54 (0.42–0.65) compared with 0.68 (0.50–0.86) in those not requiring dialysis or 0.65 (0.56–0.73) in those with a kidney transplant.^49^ Social interaction reporting saw 30% (*n* = 24) having no social interactions in a week, 53% (*n* = 42) having 1–3 interactions and 17% (*n* = 14) having 4 or more social interactions.

### 3.2 Out‐of‐pocket expenditure

Out of pocket expenses including the source of expenditure are summarized in Table 2. The quarterly mean total out‐of‐pocket expenses reported by 80% of participants was 1264 AUD (Range 0 to $12 500), or 5056 AUD (Range 0 to $50 000) annually for these households. The highest individual quarterly expenses for these patients were transport related at a mean quarterly cost of 308 AUD (range 0 to $4000), followed by medication expenses at 240 AUD (range 0 to $2000) and health specialist fees at 142 AUD (Range 0 to $3300).

**TABLE 2:** Out of pocket expenses for 3 months (AUD).

	Mean (SE)	Range	Median	IQR
Transport	308 (73)	0 to 4000	100	300
Medication	240 (39)	0 to 2000	160	270
Specialist	142 (53)	0 to 3300	0	90
Home care	116 (43)	0 to 3000	0	0
Hospital	113 (60)	0 to 3500	0	0
Accommodation	102 (42)	0 to 2000	0	0
Special food	70 (18)	0 to 700	0	0
Diagnostic tests	54 (24)	0 to 1500	0	0
General practitioner	34 (10)	0 to 500	0	0
Ambulance	30 (24)	0 to 1860	0	0
Home mods	13 (13)	0 to 1000	0	0
Total	1264	0 to 9760	550	1260

**FIGURE 1:**
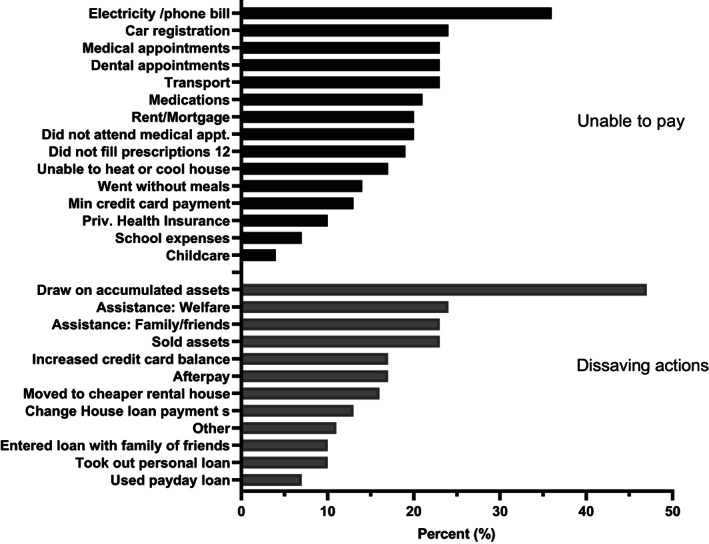
Total 3‐month self‐reported out‐of‐pocket costs by modified Monash classification.

Mean quarterly out‐of‐pocket costs by rurality (Modified Monash Classification (MMC)) are shown in Figure 1. The highest expenses were incurred by those in MMC 5 or small rural towns, and the lowest in MMC 6–7, though further research is required due to a low number of responses for those areas. Financial catastrophe, where out‐of‐pocket costs exceed 10% of family household income was seen in 54% of households.

### 3.3 Financial hardship

**FIGURE 2:**
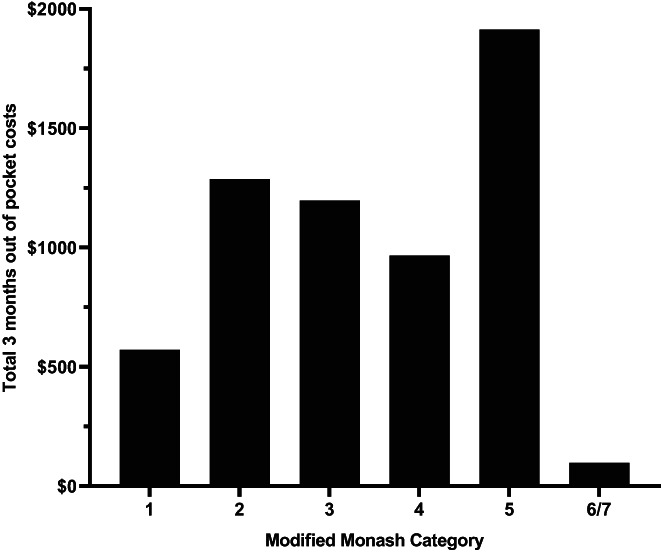
Proportion of participants reporting each financial hardship indicator (MM2‐7).

Seventy eight percent of participants (*N* = 68) reported financial hardship in the previous 12 months (Figure 2). The inability to pay the cost of electricity, gas and telephone bills placed the greatest burden for 36% (*n* = 25) of households. Inability to pay car registration was experienced by 24% (*n* = 17) of the households and 23% (*n* = 16) were unable to pay for medical appointments, dental appointments, and transportation costs. The main dissaving strategy reported was drawing on long term savings, including superannuation, by 47% of the participating households. Self‐reported prosperity or poverty reported in Table 3 was consistent with financial hardship with 75.6% of those experiencing hardship reporting as just getting on, poor or very poor, compared to 33.4% of those not experiencing financial hardship. The quarterly out‐of‐pocket costs for those who experienced hardship were higher at 1509 AUD (95% Confidence Interval (CI) $870–$2146) compared with those reporting no hardship with expenses at a much lower $918 (CI $93–$1743), however the differences were not statistically significant (*p* = .36) (Table 3). Of note, in Table 3, the proportion of participants who were or were not bulk billed (i.e., no co‐payment required) by general practitioners and specialist was the same or similar for both the hardship and non‐hardship groups.

**TABLE 3:** Out of pocket expenditure and catastrophic spending.

	No hardship *n* = 15	Hardship *n* = 53	*p*‐value
Mean (SD?) Out of pocket spending (AUD/3 months)	$918 (559)	$1509 (297)	.36
	*n* (%)	*n* (%)	
Proportion with catastrophic spending (OOP > 10% income)	10 (69.0)	32 (86.5)	.096
Income negatively impacted by CKD (yes)	9 (60.0)	48 (90.6)	.011
Private health insurance (yes)	9 (60.0)	20 (37.7)	.148
Ability to pay $2000 in a week (access to financial resources)			<.001
No	1 (6.7)	31 (55.4)	
Yes	14 (93.3)	20 (35.7)	
Do not Know	0 (0.0)	5 (8.9)	
How are you getting on financially			.02
Prosperous	2 (13.3)	1 (1.9)	
Reasonably comfortable	8 (53.3)	12 (22.6)	
Just getting on	4 (26.7)	34 (64.2)	
Poor	1 (6.7)	3 (5.7)	
Very Poor	0 (0.0)	3 (5.7)	
Are you bulk billed? (Yes)			
General Practitioner	13 (86.7)	37 (75.5)	.489
Kidney Specialist	13 (86.7)	40 (85.1)	1
Other specialists	3 (30.0)	12 (38.7)	.72

### 3.4 Distances travelled to health services

Table 4 shows mean one‐way distances for each MMC of rurality for access to nephrologists, dialysis centre, home dialysis training centres and transplantation centres and shows a significant relationship between greater distances being required to travel with more remote classifications of rurality. For dialysis and transplantation centres we did not achieve a sample size large enough to determine mean distances travelled in very remote communities (MMC7). Mean distances to access health services for all rural and remote classifications was greater than 50 kilometres for specialist nephrology services and greater than 300 kilometres for transplanting centres. Seventy four percent (74%) of respondents used their own car to travel to their dialysis centre.

**TABLE 4:** Mean (SD), kilometres travelled to access services by remoteness category (modified Monash category).

Remoteness category	*N*	General practitioner	Kidney specialist	Dialysis or training centre	Transplantation centre
MM1 metropolitan	9	17.0 (15.2)	25.6 (16.1)	24.7 (20.1)	20.4 (16.3)
MM2 regional centres	6	18.2 (17.8)	124.7 (128.1)	26.0 (28.4)	329.5 (340.3)
MM3 large rural town	20	9.9 (12.7)	57.3 (90.5)	19.3 (24.7)	362.2 (207.0)
MM4 medium rural town	17	23.7 (36.5)	202.0 (171.1)	106.6 (180.7)	281.6 (204.7)
MM5 small rural town	33	25.3 (54.4)	174.8 (145.0)	188.1 (220.5)	450.2 (221.4)
MM6 remote communities	4	6.5 (4.4)	254.2 (376.6)	334.7 (397.7)	833.2 (225.0)
MM7 very remote communities	3	36.0 (35.3)	178.0 (32.5)	NA	NA
*p* value		.81	.016	.012	<.001

*Note*: *p* value relates to differences across MMC with travel to access medical care.

## 4 DISCUSSION

In this study of rural patients with CKD in Australia, the majority (78%) experienced financial hardship and the annual mean out‐of‐pocket expenses was 5056 AUD, excluding private health insurance premiums. Almost half of participants drew on accumulated savings to be able to meet living costs, and 54% of households were classified as experiencing financial catastrophe with their out‐of‐pocket costs on healthcare being greater than 10% of their equivalized household income. Financial hardship is reflected through self‐report of inability to pay for medical care (26%), dental care (25%), medication (23%), with 22% reported missing medical appointments and 21% went without medications as they were unable to pay for them. Patients with financial hardship experienced greater out‐of‐pocket costs, with a greater proportion being classed as catastrophic spending than those without hardship. This combined with our previous qualitative work regarding patient perspectives on access, which included ‘compounding economic consequences’ and ‘unrelenting financial strain’, provide us with the subjective components of financial toxicity being experienced by rural people with CKD.^16^

Transport to health services is a major barrier for rural patients with CKD and was the largest contributor to out‐of‐pocket costs in this study.^16^, ^26^ Rural patients have fewer transport options, reduced incentre dialysis services and greater distances to access home dialysis training and transplantation services than their metropolitan counterparts.^16^, ^26^, ^50^ Importantly, the mean distance for all rural participants was greater than 50 km for nephrology services, and over 300 km for transplanting centres, and 24% had to relocate for more than 3 months to access services. The current models of health service delivery for kidney replacement therapy for rural Australians place the burden with patients to travel to centralized health services, particularly in transplantation as highlighted in Table 4, with a hub‐and‐spoke model of care.^51^ Throughout COVID‐19 we have seen an increased utilization of telemedicine for appointments for kidney doctors and for transplant recipients, which has enabled rural patients to decrease travel and maintain optimal care with their treating teams.^52^, ^53^ Nonetheless, given the particularly high impact on rural patients with CKD, alternate models of care that remove the burden of transport from patients using appropriate reimbursement policies is required to ensure equitable access.

The prevalence of financial hardship within this sample is 78%, which is slightly higher than a previous metropolitan based study of CKD at 71%,^37^ and much higher than estimated for the general Australian population at 29%.^41^ The out‐of‐pocket costs experienced annually per household in this study of 5056 AUD, is higher than the estimated average annual health expenditure of 4052 AUD per Australian household, noting this figure also includes private health insurance premiums in the order of $1500 to $2500 per year,^41^ which our study does not. In a study of out‐of‐pocket costs for cancer patients in rural Australia, the mean out‐of‐pocket costs for a median period of 21 weeks after diagnosis was 2179 AUD (CI $1873–$2518) inclusive of private health insurance premiums.^54^ These figures are similar to our study, however the sustained frequency and distances required to access dialysis for years at a time require further research into the financial toxicity experienced over time by patients with CKD.

This study confirms the impacts of financial toxicity, quality of life and the presence of chronic illness, however raises questions as to why people in rural areas with CKD report low quality of life compared to the metropolitan cohort reported by Essue et al.^37^ Overall scores for the rural participants are lower, but particularly for those currently receiving dialysis at 0.54. While the CKD‐stage specific utility scores reflect the expected improved quality of life experienced by patients receiving a transplant, they do also raise further questions as to how to improve and maximize the quality of life of rural patients whilst on dialysis and minimize the impact of financial toxicity. The high rates of depression and anxiety reported by rural patients as a comorbidity (30%) with only hypertension scoring higher at 53% are consistent with the low quality of life scores. Also patients on dialysis find it difficult to maintain employment due to symptom burden which has further negative affect on financial security, overall quality of life and independence.^55^ This is compounded for rural and people who have a background of relative educational disadvantage and high unemployment rates in these areas.^32^, ^56^

We acknowledge that this study was limited to English‐speaking participants and Australia is a high‐income country with universal health coverage which limits transferability of results to middle or low‐income settings. The numbers of respondents were limited in remote and very remote communities, and as such this is quite a targeted sample for rural patients. We measured self‐reported healthcare expenditure at one point in time and this may not reflect the true costs or hardship as it relies on participant recollection. We used an online survey so it may have limited participation to those with access to a computer or mobile phone. There are also stages of the CKD journey that may require a greater financial outlay than others for rural patients such as training for home dialysis, time of transplantation and work up for transplantation. A prospective study capturing real costs to the patients and their families including lost wages, and subjective impacts would show the true financial toxicity experienced by rural patients at crucial times in the CKD journey. A future study with increased numbers of participants from remote communities would be beneficial, together with how financial toxicity experienced impacted decisions regarding choices for different modalities of kidney replacement therapy. It would be important to explore perceptions and awareness of the healthcare providers of the impacts of financial hardship on patient's care, decision making and ability to maintain treatment options.

Innovative models of care are required to address financial burden, particularly the out‐of‐pocket expenses which are carried by those who can afford it the least, if the objectives of universal healthcare provision in Australia are to be met.^33^ The health service costs of providing dialysis services is highest in remote communities.^57^ One example of innovation in Australia is the Purple House dialysis services and mobile trucks that provide essential services to some of the remotest communities in Central and Northern Australia through the Western Desert Nganam pa Walytja Palyantjaku Tjutaku Aboriginal Corporation.^58^ This is partially subsidized through a Government activity based service reimbursement following advocacy from remote Indigenous leaders, as well as philanthropic support and other Government grants, to cover the expenses of those receiving treatment and aims to ‘help them to have the best life they can’.^58^, ^59^ Further research should explore whether these types of innovative service models can be adapted to rural, as well as remote settings.

## 5 CONCLUSION

Rural households experience considerable financial toxicity due to out‐of‐pocket costs associated with accessing CKD and other health issues related care despite living in a high‐income country with universal healthcare available. A large percentage of these costs were travel and transport related. Over half of rural households with CKD in this study required access to their savings or superannuation to afford living costs and experienced financial catastrophe. A greater understanding of the impact of financial toxicity experienced by rural households with CKD and the impacts this has on their treatment choices, health outcomes and quality of life is needed, particularly given the lifelong requirement that people with CKD have for access to specialist health care services.

## AUTHOR CONTRIBUTIONS

Research idea and study design: Nicole Scholes‐Robertson, Martin Howell, Talia Gutman, Allison Tong, Beverley M. Essue, Germaine Wong, Jonathan C. Craig; data acquisition: Nicole Scholes‐Robertson, Martin Howell; data analysis/interpretation: Nicole Scholes‐Robertson, Katrina Blazek, Jonathan C Craig, Allison Tong, Martin Howell; supervision or mentorship: Martin Howell, Katrina Blazek, Beverley M. Essue, Kirsten Howard, Germaine Wong, Jonathan C. Craig, Allison Tong. Each author contributed important intellectual content during manuscript drafting or revision and accepts accountability for the overall work by ensuring that questions pertaining to the accuracy or integrity of any portion of the work are appropriately investigated and resolved.

## FUNDING INFORMATION

NSR is supported by a National Health and Medical Research Council Postgraduate Scholarship (ID1190850). AT is supported by a National Health and Medical Research Council (NHMRC) fellowship (1106716). The funding organizations had no role in the design and conduct of the study; collection, management, analysis, and interpretation of the data; preparation, review, or approval of the manuscript.

## CONFLICT OF INTEREST STATEMENT

The authors have no conflicts of interest to declare.
